# A KASP Genotyping Method to Identify Northern Watermilfoil, Eurasian Watermilfoil, and Their Interspecific Hybrids

**DOI:** 10.3389/fpls.2017.00752

**Published:** 2017-05-08

**Authors:** Eric L. Patterson, Margaret B. Fleming, Kallie C. Kessler, Scott J. Nissen, Todd A. Gaines

**Affiliations:** Department of Bioagricultural Sciences and Pest Management, Colorado State University, Fort CollinsCO, USA

**Keywords:** genotyping, KASP, invasive, aquatic weed, hybridization

## Abstract

The invasive aquatic plant Eurasian watermilfoil (*Myriophyllum spicatum* L.) can hybridize with the related North American native species northern watermilfoil (*M. sibiricum* Kom.). Hybrid watermilfoil (*M. spicatum* × *M. sibiricum*) populations have higher fitness and reduced sensitivity to some commonly used aquatic herbicides, making management more difficult. There is growing concern that management practices using herbicides in lakes with mixed populations of watermilfoil species may further select for hybrid individuals due to the difference in herbicide sensitivity. Accurate and cost-effective identification of rare hybrid individuals within populations is therefore critical for herbicide management decisions. Here we describe KASP assays for three SNPs in the ITS region to genotype individuals from both parental watermilfoil species and their hybrid, using synthesized plasmids containing the respective sequences as positive controls. Using KASP we genotyped 16 individuals from one lake and 23 individuals from a second lake, giving a highly accurate picture of *Myriophyllum* species distribution dynamics. We identified one hybrid individual among 16 samples from one lake, a discovery rate of <10%. Discriminant analysis showed that while a single SNP was generally sufficient for genotyping an individual, using multiple SNPs increased the reliability of genotyping. In the future, the ability to genotype many samples will provide the ability to identify the presence of rare individuals, such as a less common parental species or the inter-specific hybrid. Lakes with complex species distribution dynamics, such as a low proportion of hybrids, are where herbicide application must be carefully chosen so as not to select for the more vigorous and less herbicide-sensitive hybrid individuals.

## Introduction

The invasive aquatic plant Eurasian watermilfoil (*Myriophyllum spicatum* L.) was introduced to the United States from Asia during the 1940s (Couch and Nelson, 1988; Moody et al., 2016). After introduction, this submersed species spread rapidly throughout the USA, forming dense monotypic mats that have caused economic and ecological damage to infested lakes, streams, and reservoirs (Eiswerth et al., 2000; Olden and Tamayo, 2014). The decrease in native plant diversity that occurs after *M. spicatum* invasion is an alarming ecological impact (Madsen et al., 1991).

Like many aquatic plants, *M. spicatum* is a perennial that often reproduces asexually, a strategy that has the advantage of cloning better-adapted genotypes in stable environments (Philbrick and Les, 1996). *M. spicatum* uses a very simple form of asexual reproduction called autofragmentation. Autofragmentation usually occurs soon after flowering when stems that are relatively fragile break off from the parent plant and float away as structures that can start new colonies (Grace, 1993). One characteristic of these shoot pieces that adds to the success of autofragmentation is the development of adventitious roots. *M. spicatum* has retained the ability to reproduce sexually using a common strategy of many aquatic angiosperms. Modified stems project from the vegetative mat and terminate in monecious flowers above the water surface. This allows for flowers to be wind-pollinated. Sexual reproduction and subsequent seed production are often overlooked as an important survival strategy for *M. spicatum*. Seed production can be an insurance against local extinction because there is some seed dormancy (Grace and Wetzel, 1978). Sexual reproduction can also produce new genotypes that could be better adapted to changing environments.

Environmental change for aquatic plants is usually thought of in terms of salinity, pH, or turbidity, but not necessarily annual applications of aquatic herbicides. It is now apparent that *M. spicatum* has hybridized with the related North American species northern watermilfoil (*M. sibiricum* Kom.) (Moody and Les, 2007; Zuellig and Thum, 2012; Grafe et al., 2015). In the absence of herbicide selection pressure, hybrid watermilfoil (*M. spicatum* × *M. sibiricum*) populations appear to have higher fitness, manifested as a faster and more aggressive growth rate than either parental species in both laboratory and field conditions (LaRue et al., 2013; Hovick and Whitney, 2014). Some hybrid populations are also less sensitive to several commonly used aquatic herbicides, including 2,4-D, fluridone, norflurazon, and topramazone (LaRue et al., 2013; Berger et al., 2015). There is growing concern that current management practices in lakes with mixed populations of watermilfoil species, which rely heavily on herbicide application, may further select for hybrid populations due to the difference in herbicide sensitivity.

Several methods to accurately identify *M. spicatum*, *M. sibiricum*, and *M. spicatum* × *M. sibiricum* hybrid individuals using morphological characteristics have been proposed. Morphological characteristics, while sufficient to distinguish between *M. spicatum* and *M. sibiricum*, are no longer reliable once hybrid individuals are present, as the hybrid characteristics are often intermediate between the two species (e.g., the number of pinnae or leaflet pairs) (Coffey and McNabb, 1974; Moody and Les, 2007).

Sufficient genetic variation exists between the two species that genotyping is an accurate method for species identification (Moody and Les, 2002; Sturtevant et al., 2009). Current methods rely on 23 intra-genic polymorphic single nucleotide polymorphisms (SNPs) within the first and second nuclear ribosomal internal transcribed spacer regions (ITS1 and ITS2) of *M. spicatum* and *M. sibiricum* (Moody and Les, 2002). Of these SNPs, 11 clearly distinguish between *M. spicatum* and *M. sibiricum*. When a single individual is heterozygous for both alleles of a single SNP, it indicates the individual is an inter-specific hybrid. A hybrid individual will also be heterozygous for the remaining 10 SNPs due to linkage of the SNPs within the ITS regions.

Single nucleotide polymorphism genotyping in these species has been performed using several methods. Originally, the ITS region was amplified via polymerase chain reaction (PCR), the PCR products were cloned, and multiple clones were sequenced to determine their genotype (Grafe et al., 2015). This process requires the longest time and highest cost per sample of available methods. Subsequently, genotyping was streamlined with the development of a PCR restriction fragment length polymorphism (PCR-RFLP) assay using either a *Bmt*I or *Fsp*I restriction digest that cut at base pair (bp) 274 or 551 of the ITS amplicon, respectively. By eliminating the cloning and sequencing for species identification with the PCR-RFLP assay, Grafe et al. (2015) were able to substantially decrease the amount of time and money per sample required for positive species identification of individual watermilfoil specimens. The higher throughput enabled larger sample sizes, providing a more accurate estimate of *Myriophyllum* species distribution dynamics.

Recent advances in SNP genotyping provide more cost-effective and accurate results than PCR-RFLP. Currently, the Kompetitive Allele Specific PCR (KASP) assay is a common technique for genotyping SNPs. This assay is used in several fields of study, including plant breeding, disease identification, and species identification (Semagn et al., 2014). KASP is able to discriminate between two alleles of a SNP using a common reverse primer paired with two forward primers, one specific to each allele. Each forward primer also has a nucleotide sequence that hybridizes to either the HEX or FAM fluorophore quencher. Amplification proceeds using stringent conditions to permit forward primers to bind only if they are perfectly complementary to the template sequence. Fluorophores are released from the quencher molecule when a forward primer is incorporated in a PCR product, causing the released fluorophore to fluoresce. This fluorescence is detected at the end of the assay using a real-time PCR machine, and the proportion of fluorescence from HEX, FAM, or both indicates the genotype of the sample.

Kompetitive Allele Specific PCR genotyping has several advantages compared to other genotyping assays. KASP assays are more convenient, as thels can be genotyped simultaneously on a standard 96-well plate, giving a much more accurate view of the *Myriophyllum* species distribution dynamics, and increasing the likelihood of detecting a rare hybrid individual. KASP assay design is very flexible, as useful SNPs are not limited to available restriction enzyme recognition sites, and primers can even cover stretches of sequence containing multiple SNPs by incorporating degenerate or mixed bases into the primer sequence. KASP assays are quantitative and therefore amenable to statistical analysis, such as assigning probabilities to genotyping calls. Data from multiple SNP genotyping assays can be integrated into a single model, increasing the robustness of species diagnostics.

Here we describe KASP assays for three SNPs in the ITS region to genotype individuals from both parental watermilfoil species and their hybrid, using synthesized plasmids containing the appropriate sequences as positive controls. Using these three KASP assays, we genotyped 23 and 16 individuals, respectively, from two lakes, giving a highly accurate picture of *Myriophyllum* species distribution dynamics in each case. Discriminant analysis showed that while a single SNP was generally sufficient for genotyping an individual, using multiple SNPs increased the reliability of genotyping.

## Materials and Methods

### Plant Collection

Several previously identified *M. spicatum* biotypes and known inter-specific watermilfoil hybrid (*M. spicatum* × *M. sibiricum*) biotypes (eight biotypes each) were harvested from aquaponics cultures maintained in the CSU Weed Research lab. Unknown *Myriophyllum* individuals were collected from the following two lakes in northern Colorado: Rainbow Lake located at 40.506758, -104.989224, and Walleye Lake at 40.505680, -104.982883. Individual stems (Rainbow, *n* = 23; Walleye, *n* = 16) were collected from each lake by rake throws. A single leaf from a stem was used for extraction and therefore a tissue sample is expected to represent a unique individual. Tissue samples were stored in sealed bags with damp paper towels at 4°C until DNA extraction.

### Plant DNA Extraction

DNA was extracted from 50 mg of watermilfoil leaf tissue using a modified CTAB method (Doyle, 1991). All steps were performed at room temperature (22°C) unless otherwise indicated and all chemicals were of molecular biology grade. In brief, tissue was initially ground to a fine powder with a metal bead in 500 μL of 2x CTAB buffer (2% CTAB, 1% PVP, TRIS-EDTA pH 5) using a TissueLyser II (Qiagen, Germantown, MD, USA) at 30 oscillations/second for 1 min. Ground samples were incubated at 65°C for 1 h, after which 500 μL of phenol:chloroform:isoamyl alcohol (25:24:1) was added. The samples were slowly rocked on an orbital shaker for 15 min. Samples were centrifuged at 10,000 × *g* for 5 min. The upper phase was transferred to a new tube, to which 500 μL of chloroform:isoamyl alcohol (24:1) was added. The samples were again centrifuged at 10,000 × *g* for 5 min. The upper phase was transferred to a new tube and nucleic acids were precipitated using 0.1 volumes of 3 M sodium acetate and 2.5 volumes of 100% ethanol. Samples were precipitated at 4°C for 15 min and then centrifuged at 15,000 × *g* for 15 min. The resulting pellets were re-suspended in 50 μL of sterilized water. DNA concentrations and quality were assessed using a spectrophotometer (NanoDrop 2000 Spectrophotometer, Thermo Fisher Scientific, Wilmington, DE, USA). Samples were subsequently diluted to 5 ng/μL for use in all KASP assays.

### Plasmid Design

**FIGURE 1:**
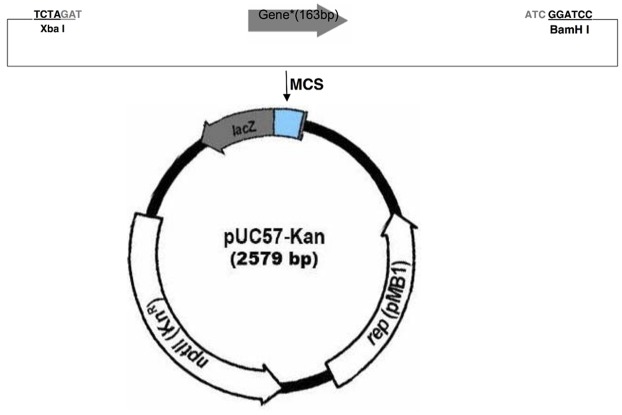
**Plasmid map illustrating the cloning strategy for the two plasmid inserts in the pUC57-Kan plasmid (GenScript).** The cloning strategy and insert size are identical for the two plasmids, so a generic map is given that represents the strategy for both plasmids.

Two plasmids were designed as positive controls for the KASP assay. Plasmid inserts consisted of the sequence within the ITS region complementary to the genotyping primers, with all inter-primer sequence removed (**Figure 1** and **Table 1**). The complete oligonucleotides were synthesized by GenScript (Piscataway, NJ, USA) in the pUC57-Kan plasmid.

**Table 1:** The full sequence of the plasmid inserts for *M. sibiricum* and *M. spicatum* positive plasmid controls.

Plasmid	Gene name	Length (bp)	Vector name	Sequence (5′-3′)
1	M_Sib_Positive_Control	163	pUC57-Kan	CATGACGAACTTAGCACACCGCTAGCCGACTTGTGCGGCAGCGGCGTTGCAAACTTCGATACCTA
				CAAAGCCCACCCTTCAAGGATATGGTGCTGCGGAAGCAGATATTGGATAACTCAGCCTTTGTTGCG
				TCGTGCCCGCCGTGCCCCTTGGAGCTCAGCAT
2	M_Spi_Positive_Control	163	pUC57-Kan	CATGACGAACTTAGCACACCACTAGCCGACTTGTGCGGCAGCGGCGTTGCAAACTTCGATACCTA
				CAAAGCCCACCCTTCAAGGATAAGGCGCTGCGGAAGCAGATATTGGATAACTCAGCCTTTGTTGCG
				CCGTGCCCGCCGTGCCCCTTGGAGCTCAGCAT


Control plasmids were transformed into DH5α *E. coli* cells using a standard heat transformation protocol (provided by GenScript) and selection on LB plates using 50 μg/mL kanamycin. Individual colonies were transferred to a numbered patch plate and allowed to grow at 37°C for 16 h.

### *E. coli* DNA Extraction

DNA was extracted from cultures grown from ten colonies on each patch plate. A toothpick was dipped into the *E. coli* colony and used to inoculate 1 mL of LB containing 50 μg/mL kanamycin. After incubating for 16 h at 37°C with shaking, the *E. coli* cultures were pelleted by centrifugation at 8,000 × *g*. DNA was extracted from the pellets using Qiaprep Spin Miniprep kit (Qiagen, Germantown, MD, USA) according to the manufacturer’s recommendations. DNA concentrations and quality were assessed using a NanoDrop 2000 spectrophotometer. Extracted plasmids were subsequently diluted to 5 pg/μL for use in all KASP assays. A 1:1 mixture of the diluted plasmids was used in KASP assays to simulate an inter-specific hybrid.

### Primer Design

Three primer sets were designed for the KASP assay to distinguish three diagnostic SNPs at bp 118, 363, and 478 in the Internally Transcribed Spacer (ITS) region (Moody et al., 2016; **Table 2**). For each primer set, the forward primer for *M. spicatum* was appended at the 5′ end with the sequence complementary to the HEX fluorophore quencher, while the forward primer for *M. sibiricum* was appended at the 5′ end with the sequence complementary to the FAM fluorophore quencher. The forward primers for *M. sibiricum* at SNPs 118 and 478 spanned sequences containing SNPs that discriminate between sub-populations, which required the use of degenerate bases in the primers. Degenerate bases are indicated according to the universal IUPAC code (Cornish-Bowden, 1985). The forward primers for SNPs 118 and 478 in *M. sibiricum* designed to amplify these degenerate bases were an equal blend of the two possible alleles at the degenerate SNP.

**Table 2:** Kompetitive Allele Specific PCR primers for SNPs 118, 363, and 478 in the *Myriophyllum* ITS region.

Primer name	Primer sequence (5′-3′)	OligoAnalyzer 3.1 predicted melting temperature
**SNP 118 (G/A)**		
*M. sibiricum* FP-118	CATGACGWACTTAGCACACCG	55.9°C
*M. spicatum* FP-118	CATGACGAACTTAGCACACCA	55.2°C
Universal RP-118	TAGGTATCGAAGTTTGCAACGC	55.5°C
**SNP 363 (A/G)**		
*M. sibiricum* FP-363	CAATATCTGCTTCCGCAGCA	55.6°C
*M. spicatum* FP-363	CAATATCTGCTTCCGCAGCG	56.6°C
Universal RP-363	CAAAGCCCACCCTTCAAGGA	57.7°C
**SNP 478 (T/C)**		
*M. sibiricum* FP-478	GATAACTCAGCCTYTGTTGCGT	56.4°C
*M. spicatum* FP-478	GATAACTCAGCCTTTGTTGCGC	56.9°C
Universal RP478	ATGCTGAGCTCCAAGGGGCA	61.8°C
5′ FAM TAG (*M. sibiricum*)	GAAGGTGACCAAGTTCATGCT	
5′ HEX TAG (*M. spicatum*)	GAAGGTCGGAGTCAACGGATT	


### KASP Assay

A primer master mix including forward and reverse primers for a single SNP was made according to the KASP assay manufacturer’s recommendations (LGC Genomics, Beverly, MA, USA). After all primers were re-suspended in Tris-HCl, pH 8.3, at 100 μM, a primer master mix was assembled with 18 μL of the *M. spicatum* forward primer, 18 μL of the *M. sibiricum* forward primer, 45 μL of the common reverse primer, and 69 μL of 10 mM Tris-HCl, pH 8.3. KASP master mixes were made for each SNP assay with 432 μL LGC Genomics Master Mix (which includes polymerase, dNTPs, buffer, and HEX- and FAM-tagged oligonucleotides) and 11.88 μL of the appropriate primer master mix.

Kompetitive Allele Specific PCR reactions were assembled in a 96-well plate with 4 μL of master mix and either 4 μL water (no-template control), 4 μL genomic DNA at 5 ng/μL, or 4 μL of plasmid DNA at 5 pg/μL. Reactions were performed in a Bio-Rad CFX Connect (Bio-Rad Laboratories, Inc., Hercules, CA, USA) according to the following standard KASP PCR program: activation at 94°C for 15 min, then 10 touchdown cycles of 94°C for 20 s (denaturing), 61–55°C for 60 s (dropping 0.6°C per cycle, for annealing and elongation), 23°C for 30 s (to permit accurate plate reading), followed by 26 cycles of 94°C for 20 s, 55°C for 60 s, 23°C for 30 s. Fluorescence was tracked in real-time with plate reads at the end of every amplification cycle. Fluorescence data from the cycle showing the greatest distinction between clusters without any background amplification was used for genotyping, which was determined to be cycles 22–24 of the amplification phase.

### Data Analysis

Due to slight variations in maximum fluorescence and fluorescence in the no-template controls between plates, HEX and FAM fluorescence for each data point were transformed as a percentage of the maximum fluorescence for each fluorophore within a plate. Maximum fluorescence is defined as the highest FAM or HEX signal from any reaction in a 96-well plate. Cutoffs for genotyping calls on unknown samples were drawn by calculating the point halfway between the mean (*x*,*y*) coordinate of the control (plasmid) hybrid and either the control *M. sibiricum* or *M. spicatum* clusters, then drawing a line from that point to the origin (0,0). Additionally, a zone of “no amplification” was defined as 30% based on the maximum fluorescence observed in no-template controls. The 30% cut-off was selected because technical variation at low fluorescence values is more likely to influence genotyping calls than at high fluorescence values, and based on the observation that none of our no-template controls exceeded 30%. A quarter circle at 30% around the axis intercept was used to define this zone. Genotypes were assigned to unknown samples based on the sector of the plot where their fluorescence values occurred. If a sample fell within the bounds of a zone it was assigned that genotyping call.

Once each sample (experimental samples as well as plasmid controls) was assigned a genotype based on its zone, linear discriminant analysis was performed in JMP 12.2 (SAS Institute Inc., Cary, NC, USA) to evaluate the probability of that individual having its assigned genotype. Genotyping results from each SNP were first assessed independently, then using all three SNPs combined to provide more robust probabilities.

## Results

We developed three KASP primer sets that distinguish between the native *M. sibiricum* and the invasive *M. spicatum* species as well as inter-specific hybrids. Our KASP primers utilize the previously identified SNPs at base pairs 118, 363, and 478 of the ITS region (**Table 2**). We tested the primer sets on plasmids containing known sequences, on known lab biotypes of *M. spicatum* and hybrids, and on unknown *Myriophyllum* individuals harvested from two lakes in northern Colorado. We assigned genotypes manually, and then measured the reliability of the genotyping calls using discriminant analysis to assign probabilities to calls from each SNP individually as well as using all three SNPs together.

### KASP Assays on Plasmids

We developed two plasmids (**Figure 1**) to serve as positive controls for the KASP-PCR. Plasmid controls were ideal because they allow for rapid generation of DNA of a known genotype and eliminate the need to maintain both species of *Myriophyllum* as well as the inter-specific hybrid in hydroponic culture as positive genotyping controls.

**FIGURE 2:**
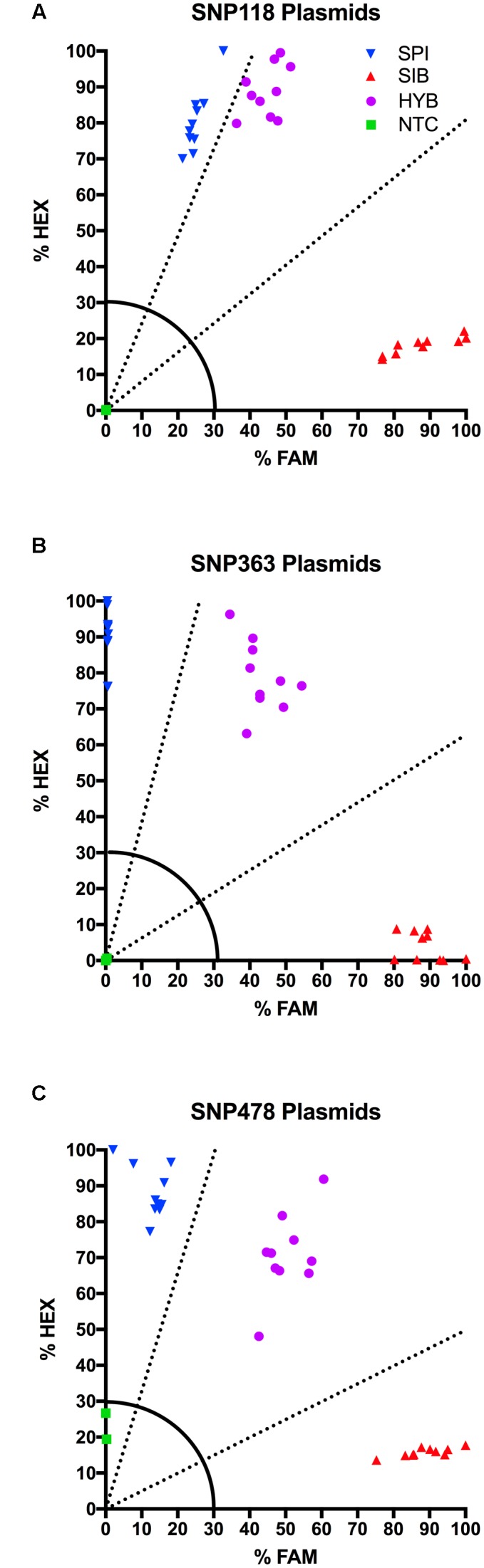
**Results of KASP assays for plasmids containing the *M. sibiricum* positive control (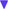), *M. spicatum* positive control (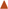), 1:1 mixture of the two to represent hybrids (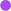), and no-template controls (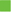).** SNPs 118, 363, and 478 are shown in panels **(A–C)**, respectively. Dashed lines represent cutoffs for making genotyping calls. The solid quarter circle line is the cutoff for no amplification.

The plasmid DNA performed consistently from assay to assay and allowed us to more accurately characterize unknown individuals in the KASP assay. For SNP 118, SNP 363, and SNP 478, we tested ten distinct *E. coli* colonies. All ten samples containing a given genotype formed a tight, distinct cluster on the HEX-FAM *x-y* plot with fluorescence values well above the 30% cut-off (**Figure 2**). SNP 118 had a very clear *M. sibiricum* cluster, but the *M. spicatum* and the 1:1 synthetic hybrids were relatively close to each other, due to increased FAM fluorescence for the *M. spicatum* samples (**Figure 2A**). However, there was no overlap between the *M. spicatum* and the synthetic hybrid sample clusters. SNP 363 and SNP 478 show obvious separation of the fluorescence signal from each of the three possible genotypes, with the *M. spicatum* plasmids having almost exclusively HEX signal, *M. sibiricum* plasmids having almost exclusively FAM signal, and the 1:1 mixture of each genotype having both HEX and FAM signals (**Figures 2B,C**). No plasmid had an ambiguous call or fell below the 30% fluorescence threshold for any of the three SNPs. This test confirmed the utility of plasmids as internal positive controls for plant genotyping assays.

### KASP Assays on Lab Biotypes

**FIGURE 3:**
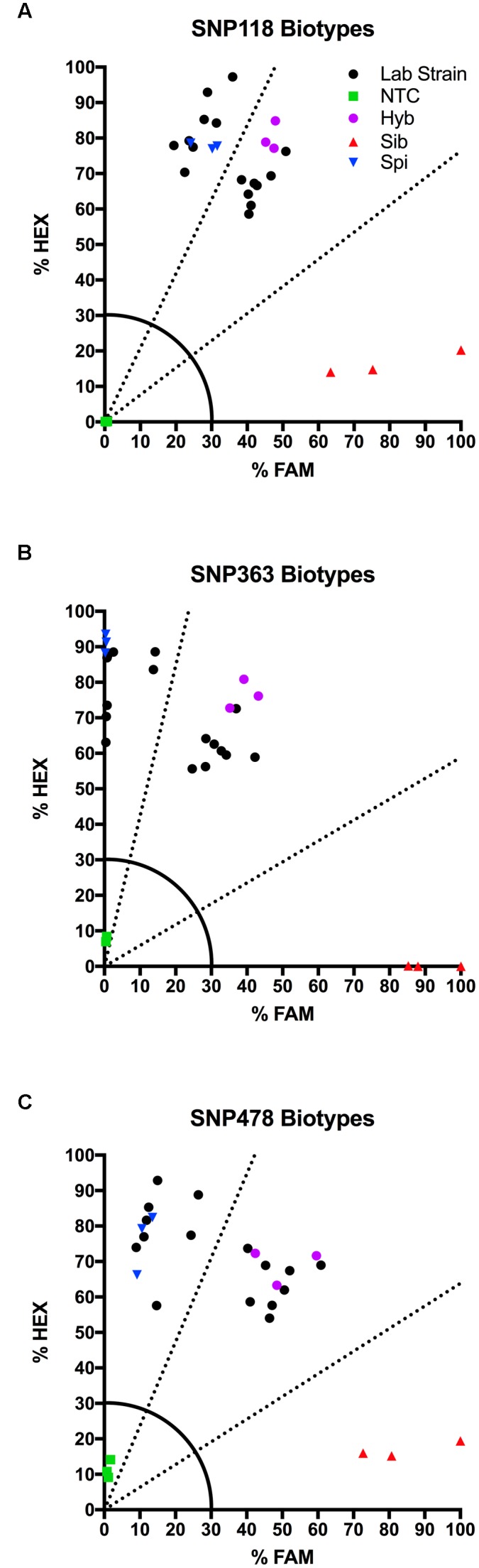
**Results of KASP assays for SNPs 118** **(A)**, 363 **(B)**, and 478 **(C)** from 16 lab biotypes (eight known inter-specific hybrids and eight known *M. spicatum* biotypes; 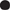), as well as the *M. sibiricum* positive control (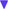), *M. spicatum* positive control (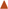), a 1:1 mixture of the two to represent hybrids (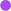), and no-template controls (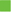). Dashed lines represent cutoffs for making genotyping calls. The solid quarter circle line is the cutoff for no amplification.

We tested several biotypes of *Myriophyllum* that are maintained in aquaponics culture at CSU. These biotypes were originally collected from various locations in North America (**Table 3**). The KASP results from all three SNP primer sets showed that eight of these biotypes clustered with the *M. spicatum* plasmid control, with high HEX signal and minimal FAM signal (Norway, CSU KCK, 4BC, St Helens, Hall, Stoney 2, Fawn, Hanbury), while eight clustered with the 1:1 synthetic hybrid mixture of *M. spicatum* and *M. sibiricum* plasmid controls, with approximately equal HEX and FAM fluorescent signals (Hayden, Mattoon, Houghton, Alpine 2, Alpine 3, Richard Farm, Jeff, Alpine 1) (**Table 3** and **Figure 3**). The genotyping calls from the KASP assay matched the known genotypes of the samples exactly.

**Table 3:** Kompetitive Allele Specific PCR SNP genotyping calls and probability of accuracy (Prob) for eight known *M. spicatum* (M. spi, dark gray) biotypes and eight known hybrid (Hyb, light gray) watermilfoil (*M. spicatum* × *M. sibiricum*) biotypes.

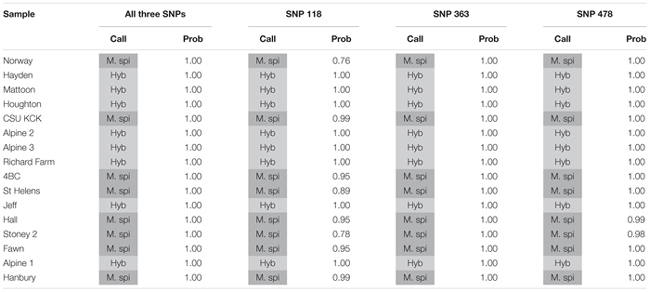

The probability that a genotype call was correct was calculated by performing discriminant analysis on the corrected fluorescence data for each SNP separately and for all three SNPs together (**Table 3**). Particularly for SNP118, several individuals had a reduced probability that the genotype was correct (e.g., Norway or Stoney 2). However, when all three SNPs were considered together, the probability was 100% for each genotype call (**Table 3**). These results confirm that all three SNPs are strongly linked and co-inherited and therefore the three SNPs can be used together to provide accurate genotyping, as would be expected for SNPs in the ITS2 region.

### KASP Assays on Rainbow and Walleye Lake

**FIGURE 4:**
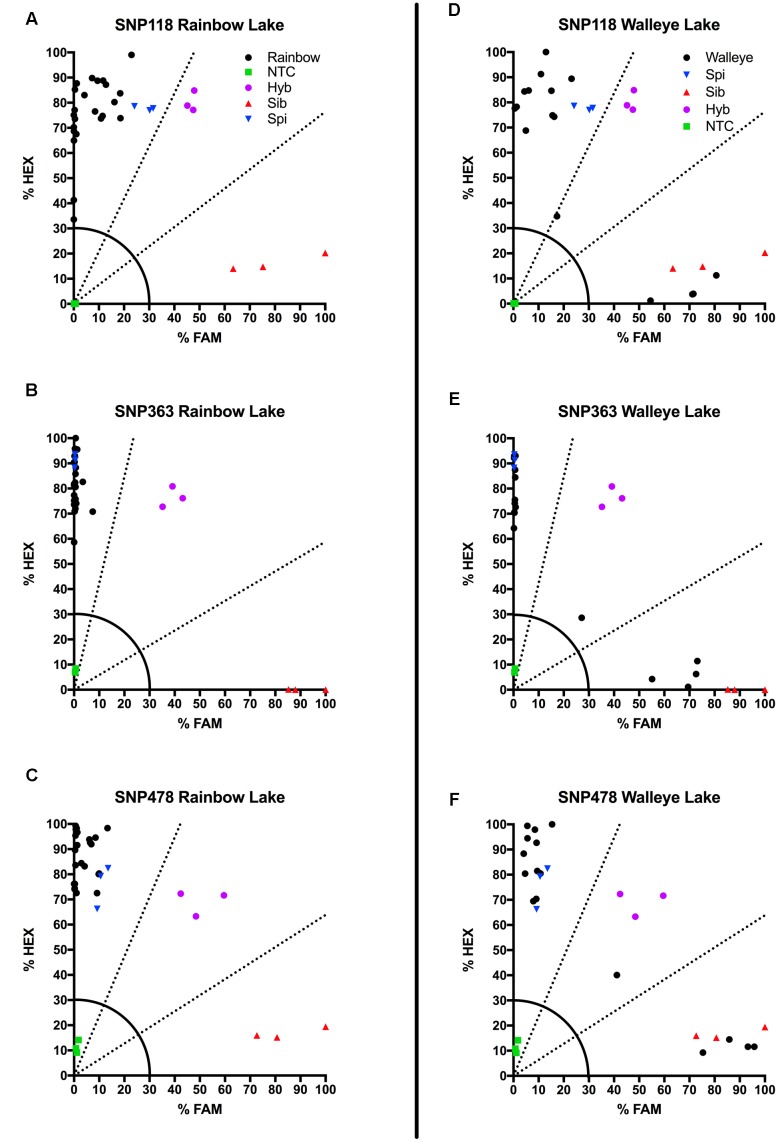
**Results of KASP assays from wild collections of unknown watermilfoil individuals from Rainbow Lake for SNP 118** **(A)**, SNP 363 **(B)**, and SNP 478 **(C)**, and from Walleye Lake for SNP 118 **(D)**, SNP 363 **(E)**, and SNP 478 **(F)**. Samples are represented as follows: *M. sibiricum* positive control (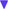), *M. spicatum* positive control (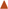), a 1:1 mixture of the two to represent hybrids (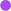), no template controls (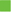), and unknown watermilfoil samples (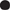). Dashed lines represent cutoffs for making genotyping calls. The solid quarter circle line is the cutoff for no amplification.

We also tested our assay on wild, unknown individuals from two lakes in northern Colorado, Rainbow Lake (*n* = 23) and Walleye Lake (*n* = 16). For Rainbow Lake, all sampled individuals were identified as the invasive *M. spicatum*, as the fluorescence signal from all three SNPs for each individual was predominantly the HEX wavelength (**Table 4** and **Figures 4A–C**). Only the genotyping call of *M. spicatu*m for plant 23 for SNP 118 was unsupported by the linear discrimination analysis (*P* = 0.085). The analysis assigned this call as “No amplification” with *P* = 0.856. However, when all three SNPs were considered together, a call of *M. spicatum* for Plant 23 was predicted with *P* = 1.0. Samples from Walleye Lake were identified as *M. spicatum*, *M. sibiricum* and inter-species hybrid genotypes, with 11 individuals showing predominantly HEX fluorescence and clustering with the *M. spicatum* plasmid controls, while four individuals (plants 2, 3, 8, and 12) showed predominantly FAM fluorescence and clustered with the *M. sibiricum* plasmid controls (**Table 5** and **Figures 4D–F**). Additionally, one individual (plant 1) was identified as the hybrid genotype, as all three SNPs showed unambiguous dual HEX and FAM fluorescence and clustered with the artificial hybrid (**Table 5** and **Figures 4D–F**).

**Table 4:** Kompetitive Allele Specific PCR SNP genotyping calls and probability of accuracy (Prob) for 23 unknown watermilfoil individuals from Rainbow Lake. M. spi (dark gray) = *M. spicatum*.

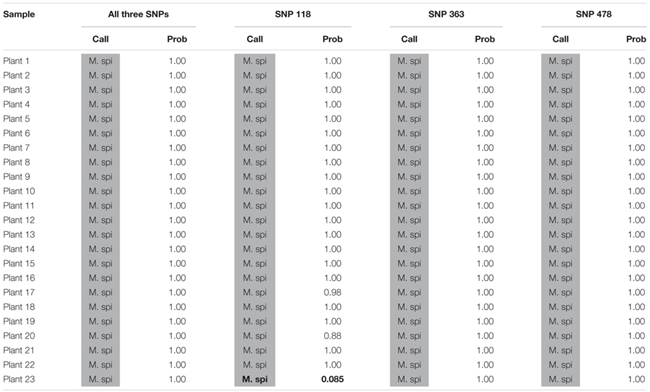

*Bolded font indicates that the genotyping call made by the point’s location does not agree with the linear discriminant analysis.*

**Table 5:** Kompetitive Allele Specific PCR SNP genotyping calls and probability of accuracy (Prob) for 16 unknown watermilfoil individuals from Walleye Lake.

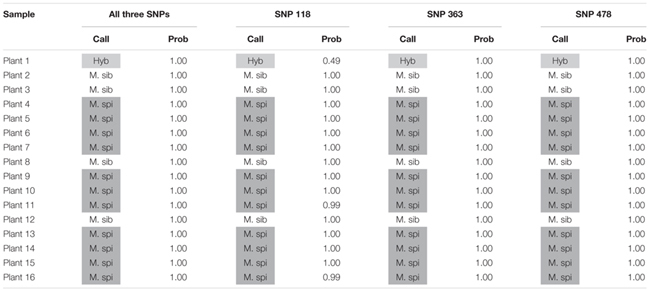

*M. spi (dark gray) = *M. spicatum*; Hyb (light gray) = inter-specific hybrid (*M. spicatum* × *M. sibiricum*); M. sib (white) = *M. sibiricum*.*

## Discussion

Discriminant analysis verified the accuracy of the genotyping calls, with SNP 118 producing a few lower-confidence genotype calls (for plants 20 and 23 from Rainbow Lake and plant 1 from Walleye Lake) but 100% probability of a correct call when data from all three SNPs were considered simultaneously. The forward primers for both SNP 118 and SNP 478 used one degenerate base each; however, the calls for SNP 478 appear to be much more accurate than for SNP 118. The degenerate bases in each case were required to allow efficient amplification of ITS sequences, because SNPs exist in the forward primers that distinguish between different sub-populations of *M. sibiricum*, but not between *M. spicatum* and *M. sibiricum*. Performance differences in the three KASP markers may be attributed to the type and location of the degenerate base. For the SNP118 forward primer, a W indicates an A or T which is either a purine or pyrimidine, respectively, while the forward primer for SNP478 contains a Y indicating a C or T, which are both pyrimidines. Degenerate bases may lower optimal annealing temperature of the primer, which in turn lowers the ability of the primer set to distinguish between our two species. The use of multiple SNPs together for genotype identification overcomes inefficiencies of any one marker, as they are always co-inherited.

The ability to genotype dozens of individuals provides the ability to identify the presence of rare individuals, such as a less common parental species or the inter-specific hybrid. Lakes with complex species distribution dynamics, such as low proportion of hybrids, are where herbicide application must be carefully chosen so as not to select for the more vigorous and less herbicide-sensitive hybrid individuals. Only recently were hybrids suspected (Moody and Les, 2002), and then determined to be more invasive and less herbicide sensitive (LaRue et al., 2013). The exact distribution, population sizes, and proportions of hybrid, *M. sibiricum*, and *M. spicatum* are largely unknown, in part due to the cost and limited throughput of current genotyping methods. The genetic diversity of the two *Myriophyllum* species in the ITS region has been explored by Sturtevant et al. (2009), including characterization of the various within-species ITS genotypes that exist. Our KASP markers are useful to distinguish between *M. spicatum*, *M. sibiricum* and their interspecific hybrid, but not to distinguish within-species genetic variation.

Monitoring has been conducted in some areas of North America (e.g., Zuellig and Thum, 2012; Grafe et al., 2015; Moody et al., 2016), but the distribution of the invasive species on the continental scale remains undetermined. With the ability to genotype hundreds of individuals rapidly and inexpensively using KASP, aquatic weed managers will be able to make more informed decisions about herbicide type and application rates, such as choosing specific herbicides and rates to control rare hybrid individuals only when they are confirmed to be present. Appropriate sampling structures would be critical, including spatially dispersed locations within a lake, to avoid redundant sampling of any clonal plants and ensuring detection of potentially rare hybrid individuals. Larger data sets comprised of accurate genotyping data will allow modeling of *Myriophyllum* species distribution dynamics, testing the hypothesis that increased selection pressure from herbicide application favors hybrid individuals due to their decreased herbicide sensitivity. Lakes can be genotyped using KASP both before and after herbicide applications to quantify shifts in species distribution dynamics toward invasive *M. spicatum* or hybrid watermilfoil individuals.

## Sources of Materials

Lake material was collected from two of the lakes at the Swift Ponds at Colorado Youth Outdoors: Rainbow Lake (40.506758, -104.989224) and Walleye Lake (40.505680, -104.982883).

## Author Contributions

EP, MF, KK, SN, and TG designed the experiments; EP, MF, and KK performed the experiments; EP, MF, and TG analyzed the results; and EP, MF, KK, SN, and TG wrote and approved the manuscript.

## Conflict of Interest Statement

EP, MF, and KK have a patent pending on the genotyping method. The other authors declare that the research was conducted in the absence of any commercial or financial relationships that could be construed as a potential conflict of interest.
